# The Mobile Insulin Titration Intervention (MITI) for Insulin Adjustment in an Urban, Low-Income Population: Randomized Controlled Trial

**DOI:** 10.2196/jmir.4716

**Published:** 2015-07-17

**Authors:** Natalie Levy, Victoria Moynihan, Annielyn Nilo, Karyn Singer, Lidia S Bernik, Mary-Ann Etiebet, Yixin Fang, James Cho, Sundar Natarajan

**Affiliations:** ^1^ Division of General Internal Medicine and Clinical Innovation Department of Medicine New York University School of Medicine New York, NY United States; ^2^ Bellevue Hospital Center New York, NY United States; ^3^ Gouverneur Diagnostic and Treatment Center New York, NY United States; ^4^ Department of Healthcare Policy and Research Weill Cornell Medical College New York, NY United States; ^5^ Division of Biostatistics Department of Population Health New York University School of Medicine New York, NY United States; ^6^ Department of Population Health New York University School of Medicine New York, NY United States; ^7^ Department of Veterans Affairs New York Harbor Healthcare System New York, NY United States

**Keywords:** patient-centered care, health care disparities, telemedicine, remote consultation, cell phones, insulin, long-acting, text messaging

## Abstract

**Background:**

Diabetes patients are usually started on a low dose of insulin and their dose is adjusted or “titrated” according to their blood glucose levels. Insulin titration administered through face-to-face visits with a clinician can be time consuming and logistically burdensome for patients, especially those of low socioeconomic status (SES). Given the wide use of mobile phones among this population, there is the potential to use short message service (SMS) text messaging and phone calls to perform insulin titration remotely.

**Objective:**

The goals of this pilot study were to (1) evaluate if our Mobile Insulin Titration Intervention (MITI) intervention using text messaging and phone calls was effective in helping patients reach their optimal insulin glargine dose within 12 weeks, (2) assess the feasibility of the intervention within our clinic setting and patient population, (3) collect data on the cost savings associated with the intervention, and (4) measure patient satisfaction with the intervention.

**Methods:**

This was a pilot study evaluating an intervention for patients requiring insulin glargine titration in the outpatient medical clinic of Bellevue Hospital Center in New York City. Patients in the intervention arm received weekday SMS text messages from a health management platform requesting their fasting blood glucose values. The clinic’s diabetes nurse educator monitored the texted responses on the platform website each weekday for alarm values. Once a week, the nurse reviewed the glucose values, consulted the MITI titration algorithm, and called patients to adjust their insulin dose. Patients in the usual care arm continued to receive their standard clinic care for insulin titration. The primary outcome was whether a patient reached his/her optimal insulin glargine dose within 12 weeks.

**Results:**

A total of 61 patients consented and were randomized into the study. A significantly greater proportion of patients in the intervention arm reached their optimal insulin glargine dose than patients in the usual care arm (88%, 29/33 vs 37%, 10/27; *P*<.001). Patients responded to 84.3% (420/498) of the SMS text messages requesting their blood glucose values. The nurse reached patients within 2 attempts or by voicemail 91% of the time (90/99 assigned calls). When patients traveled to the clinic, they spent a median of 45 minutes (IQR 30-60) on travel and 39 minutes (IQR 30-64) waiting prior to appointments. A total of 61% (37/61) of patients had appointment copays. After participating in the study, patients in the intervention arm reported higher treatment satisfaction than those in the usual care arm.

**Conclusions:**

MITI is an effective way to help low-SES patients reach their optimal insulin glargine dose using basic SMS text messaging and phone calls. The intervention was feasible and patients were highly satisfied with their treatment. The intervention was cost saving in terms of time for patients, who were able to have their insulin titrated without multiple clinic appointments. Similar interventions should be explored to improve care for low-SES patients managing chronic disease.

**Trial Registration:**

Clinicaltrials.gov NCT01879579; https://clinicaltrials.gov/ct2/show/NCT01879579 (Archived by WebCite at http://www.webcitation.org/6YZik33L3).

## Introduction

### Background

Many patients with diabetes mellitus in the United States are poorly controlled (glycated hemoglobin A_1c_ [HbA_1c_] ≥9%). This includes 48.7% of the diabetics insured by Medicaid and 27.3% of diabetics insured by Medicare [1]. The consequences of uncontrolled diabetes are severe (eg, stroke, blindness, kidney disease, and amputation) and disproportionately affect patients of low socioeconomic status (SES) [2,3]. Insulin is commonly prescribed to treat uncontrolled diabetes [4]. Patients are started on a low dose of insulin and their dose is adjusted or “titrated” according to their blood glucose levels. Adjustments are made until the patient reaches a dose that best controls their glucose levels. Insulin titration traditionally occurs during a face-to-face encounter with a clinician [5-9]. Patients show the clinician their blood glucose levels from at-home testing and then the clinician recommends an appropriate insulin titration. One titration is often not enough to achieve glycemic control, so patients need to return to the clinic for multiple appointments.

Attending multiple appointments can be challenging for low-SES patients. They are faced with competing priorities that can make the many self-care tasks of diabetes management overwhelming [10-14]. Attending a clinic appointment can mean missing work hours with an inflexible job, lost wages, copays, and arranging for childcare and transportation to the clinic. Given these challenges, the process of insulin titration and achieving glycemic control may be prolonged.

Mobile phones are increasingly used to deliver health services [15]. Research shows 84% of the low-income US population owns a mobile phone, but only 47% of this population owns a phone with advanced features (ie, smartphone) [16]. Potential health interventions designed around basic features (eg, texting and voice) would not require a smartphone and, therefore, be the most accessible for low-income populations. In addition, these technologies still allow patients and clinicians to consult one another directly, allowing for personalized, nuanced care. A recent study of smartphone apps with insulin dose calculators showed that most have significant shortcomings. These apps may not take into account the patient’s level of clinical knowledge, missing glucose readings, or concurrent oral antihyperglycemic medications, potentially introducing a safety risk [17].

### Prior Research

Studies show that short message service (SMS) text messaging is an effective medium to assist with diabetes management in general and low-SES populations [6,18-25]. It can be used successfully to remind patients to check their blood glucose levels and to gather that data so that a clinician can review it for the next in-person clinic appointment [19].

Of the few studies in which clinicians titrated insulin remotely, the interventions typically required Internet access or website navigation. Patients sent their blood glucose values to their clinicians via the Internet. Clinicians responded to these data by sending their recommendations over the Internet or by SMS text message. These studies show that it is feasible to have patients send their blood glucose data and have clinicians relay insulin dose titration advice remotely [6,22,26]. However, with our intervention we aim to show that this exchange of data can be achieved using only basic text message and voice technology.

### Current Intervention

The Mobile Insulin Titration Intervention (MITI) is a randomized controlled trial for patients who require insulin glargine titration. We chose to focus on glargine because it is the type of insulin used in our hospital formulary. Our intervention uses features available on basic mobile phones: SMS text messaging and voice calls. This technology is easy to use, low-cost, and widely available to our patient population. Through a text message, we can remind patients to check their glucose at any time and place that they have phone service. Patients can respond via text quickly and simply. Using weekly phone calls, patients and clinicians can still discuss their insulin treatment in a personal manner without the burden of an in-person appointment.

Through the MITI study, we aimed to (1) determine if MITI is effective in helping patients reach their optimal dose of insulin glargine (“optimal dose” is defined in the Intervention section), (2) evaluate the feasibility of the intervention, (3) measure the cost savings associated with the intervention, and (4) measure patient satisfaction.

## Methods

### Study Design

#### Overview

The MITI study is a randomized controlled trial that uses a parallel study design. Patients randomized to the MITI arm were allocated to receive the intervention. Patients randomized to the usual care arm acted as the control group and continued to receive standard care in the clinic. Patient outcomes were tracked for 12 weeks after enrollment in the study (see Outcome Measures section). A data and safety monitoring board reviewed any potential safety concerns for the duration of the study. This paper summarizes the methods for this trial. Further details are available in the published protocol [27].

#### Setting

This study occurred at Bellevue Hospital Center in New York City. Bellevue is part of the Health and Hospitals Corporation (HHC), the largest public hospital system in the United States. We recruited patients for this study from Bellevue’s Adult Primary Care Center (APCC). Most clinic visits are for patients who are uninsured (31%) or have Medicaid (45%) [28]. The majority of patients are nonwhite: Hispanic (41%), Black (24%), and Asian (6%) (Bellevue Hospital Center, unpublished data, 2014). The prevalence rate of diabetes in the APCC is 15% (HHC Patient Registry for Proactive Care, unpublished data, 2014), higher than the national rate of 9.3% [2].

#### Inclusion and Exclusion Criteria

The inclusion criteria for patients were initiating insulin glargine or requiring the titration of an existing insulin glargine dose, English or Spanish speaking, the most recent HbA_1c_ value at or above 8%, able and willing to inject insulin, and able and willing to provide informed consent. The exclusion criteria were patients on short-acting insulin, on systemic glucocorticoids, with sustained serum creatinine at or above 1.5 mg/dL for men and 1.4 mg/dL for women, with documented hypoglycemia unawareness, and with type 1 diabetes.

#### Recruitment and Enrollment

Patients were recruited for the study from the APCC. Clinicians referred patients to the research assistant (RA). The RA screened patients for eligibility and enrolled them in the study. The enrollment process occurred in-person in the clinic and all patients provided informed consent before participating in the study. Patients were given a US $10 Metrocard for transportation to/from the clinic and an additional US $10 Metrocard at follow-up. Patients were also given a blood glucose meter and test strips to allow them to check their blood glucose levels during the study.

#### Randomization

Patients were randomized on the day of enrollment after the informed consent process. The random allocation sequence was computer-generated by a coinvestigator and concealed in sequentially numbered envelopes. Patients were stratified by whether they were initiating insulin treatment or having their existing insulin dose adjusted. Within each stratification, the allocation sequence used blocks of 4 to help keep the number of patients balanced in each arm. Patients, clinicians, and researchers in this trial were not blinded to arm assignments.

### Intervention

After consent and randomization, patients in the MITI arm enrolled in a Web-based health management platform during the enrollment process at the clinic. The platform automatically sent patients a text message each weekday morning asking them for their fasting blood glucose value. During enrollment, the patient was able to choose English or Spanish messages and the specific time of day when the messages would be sent. When patients received the text message on their phone, they responded with their blood glucose value. The diabetes nurse educator logged onto her secure account on the platform each weekday afternoon to view the patients’ text message responses. She would call any patient that had texted an alarm value (blood glucose <80 or >400 mg/dL). Patients were instructed to call the diabetes nurse educator (which is the standard practice with patients in the clinic) in addition to sending the text if they had an alarm value.

Each Thursday afternoon, the nurse reviewed the texted values, consulted the titration algorithm (which was developed by physicians and nurses on the study team), and called the patient to adjust his/her insulin dose. The nurse could call the patient’s emergency contact on her discretion. Beginning in May 2014, we revised our study protocol and outlined voicemail as an option for the nurse to give titration instructions to patients. When the nurse was not available to check the text responses for alarm values or to make titration calls, a physician on the study team performed this task.

Patients continued with the weekday SMS text messages and weekly phone calls until they reached their optimal insulin glargine dose or for a maximum of 12 weeks. We defined optimal insulin dose as the dose at which a patient achieved at least 1 fasting blood glucose value between 80 and 130 mg/dL (inclusive) or the maximum dose that could be safely administered to the patient. During the intervention, patients continued to attend appointments with their primary care provider, but did not need to attend appointments specifically for diabetes management (eg, high HbA_1c_ clinic or diabetes nurse educator appointments). After completing the intervention, patients resumed usual clinic care. The study team arranged any follow-up appointments needed to allow the patient to resume their standard diabetes care (eg, primary care provider, diabetes nurse educator, and high HbA_1c_ clinic appointments).

### Usual Care

After consent and randomization, patients were instructed to continue with their existing treatment plan and appointments for diabetes care. After the patient had a clinic appointment for insulin titration, the RA collected data (in-person or by phone) about the appointment. These data included the patient’s insulin dose, blood glucose values, and data for cost savings outcomes.

### Follow-Up

At approximately 12 weeks after study enrollment, patients in both arms were contacted by the RA to remind them of their routine HbA_1c_ test and to ask them to fill out the Diabetes Treatment Satisfaction Questionnaire (either over the phone or when the patient was in the clinic).

### Implementation Challenges

Our initial health management platform was not able to send SMS text messages to patients with prepaid phones; thus, these patients were not able to sign up to participate in the intervention (see Participant Characteristics). These patients continued to attend in-person appointments for insulin titration and were included in the intention-to-treat analysis. Subsequent patients with incompatible phones were provided a mobile phone to use during the study. Beginning in May 2014, patients were enrolled using a different health management platform that accommodated all types of mobile phones.

We initially stratified participants by insulin treatment status (initiating insulin or needing their existing dose titrated) and by HbA_1c_ level (8%-11% or >11%). In May 2014, we decided to stratify only by insulin treatment status after finding that not all participants had an HbA_1c_ value in their medical record on the day of study enrollment.

### Outcome Measures

Our primary outcome was whether a patient reached his/her optimal insulin glargine dose within 12 weeks of enrolling in the study. We hypothesized that a greater proportion of patients in the MITI arm would reach their optimal insulin dose as compared to the usual care arm. The research staff recorded whether a patient reached his/her optimal insulin dose after each titration phone call (for the MITI arm) or after each clinic appointment (for the usual care arm). We also examined the time it took to reach optimal dose, patient self-reported hypoglycemia, and change in HbA_1c_ levels between baseline and 12 weeks.

We measured the feasibility of the intervention, including patient text response rate, the ability of the nurse to reach patients for titration phone calls, and the time the nurse spent on the intervention.

We collected data on the cost savings associated with the intervention. These data included the number and duration of insulin titration interactions (appointments during which insulin was titrated), the time patients spent traveling to the clinic and waiting prior to appointments, copays for clinic appointments, and patient health care utilization (the number of walk-in clinic, medication refill, and emergency room visits at Bellevue Hospital Center). Copays refer to the amount that the patient pays the clinic on attending an appointment with a health care provider. For patients with insurance plans, the amount is typically set by the insurance company. For uninsured patients at our hospital, the amount is based on income. For patients in our study, the most common copay was US $15.

To assess patient satisfaction with the intervention, we used the Diabetes Treatment Satisfaction Questionnaire (status version) [29]. This was administered at study enrollment and approximately 12 weeks later. We also administered the Diabetes Treatment Satisfaction Questionnaire (change version) [29] to measure the change in satisfaction after study participation. Patients in the MITI arm participated in a semistructured interview to give qualitative feedback on the intervention. This occurred when the patient reached his/her optimal dose or when the 12 weeks had elapsed.

### Statistical Analysis

Baseline characteristics were summarized using descriptive statistics and compared to determine if the arms were balanced. Chi-square tests were used for categorical outcomes and Wilcoxon rank sum tests for continuous outcomes. Interval-censoring survival analysis was used to analyze the time to reach optimal insulin dose. The generalized estimating equation (GEE) modeling was used for repeatedly measured text message responses and the duration of titration interactions. Multiple imputation was used to deal with missing data in HbA_1c_ measures. Intention-to-treat analysis was used.

## Results

### Participant Characteristics

**Figure 1:**
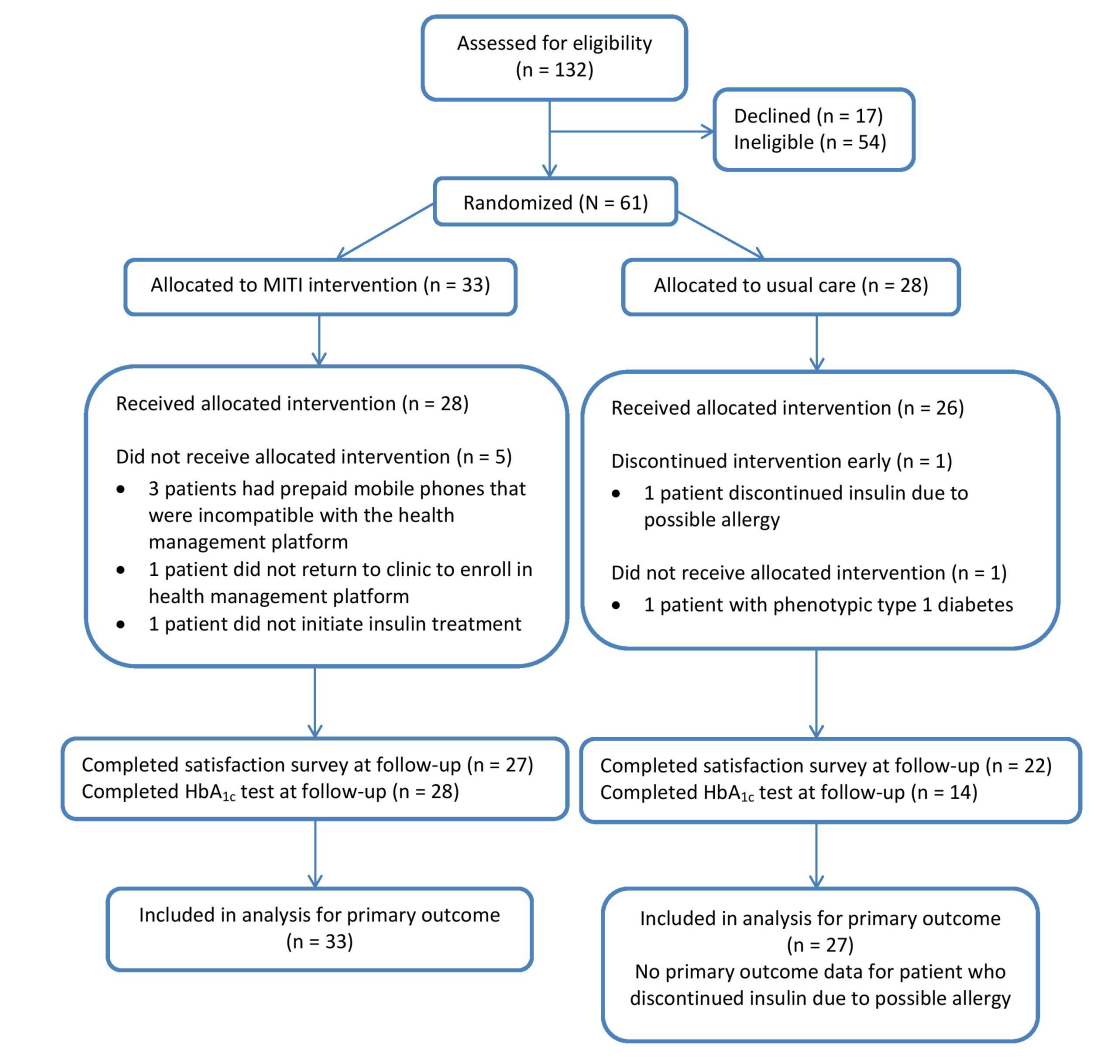
CONSORT diagram.

Patients were recruited from June 2013 to December 2013 and May 2014 to December 2014. Follow-up data were collected until March 2015. We screened 132 patients for eligibility; 54 were ineligible and 17 declined to participate (Figure 1). A total of 61 patients consented and were randomized into the study; 33 in the MITI arm and 28 in the usual care arm. There were 36 patients who were stratified as new to insulin treatment and 25 that were having their existing insulin dose adjusted. Of these 61 patients, there were 6 patients (5 in MITI and 1 in usual care) who met inclusion criteria when screened at the time of enrollment, but were discovered to be ineligible to participate soon after they consented and were randomized. Of the 5 ineligible patients randomized to the MITI arm, 3 had prepaid mobile phones that were not able to sign up for our SMS text messaging platform, 1 was not starting insulin glargine, and 1 did not return to the clinic to complete enrollment. The ineligible patient randomized to the usual care arm phenotypically fit a type 1 diabetes diagnosis. These 6 patients did not receive the allocated intervention, but were included in the intention-to-treat analysis. No significant differences in baseline characteristics/demographics were found between the 2 study arms. Demographics of participants are shown in Table 1.

**Table 1:** Demographics of participants.

Demographics		MITI (n=33)	Usual care (n=28)	Total (N=61)	*P*
Age, mean (SD)		48.48 (11.22)	44.61 (9.97)	46.70 (10.75)	.14
Gender (female), n (%)		15 (45)	16 (57)	31 (51)	.36
**Self-identified race/ethnicity, n (%)**					.85
	Hispanic	18 (55)	17 (61)	35 (57)	
	Black or African American	8 (24)	7 (25)	15 (25)	
	White	4 (12)	2 (7)	6 (10)	
	Asian	2 (6)	2 (7)	4 (7)	
	Caribbean	1 (3)	0 (0)	1 (2)	
**Highest education level,** ^a^ **n (%)**					.65
	≤Grade 8	5 (16)	8 (29)	13 (22)	
	Some high school	4 (13)	4 (14)	8 (13)	
	High school graduate/GED	13 (41)	9 (32)	22 (37)	
	Some college	4 (13)	3 (11)	7 (12)	
	College degree	2 (6)	3 (11)	5 (8)	
	Graduate degree	4 (13)	1 (4)	5 (8)	
**Annual household income (US $), n (%)**					.87
	No response	18 (55)	9 (32)	27 (44)	
	<10,000	3 (9)	5 (18)	8 (13)	
	10,000-19,999	5 (15)	6 (21)	11 (18)	
	20,000-29,999	2 (6)	4 (14)	6 (10)	
	30,000-39,999	3 (9)	3 (11)	6 (10)	
	>40,000	2 (6)	1 (4)	3 (5)	
Currently without health insurance, n (%)		23 (70)	15 (54)	38 (62)	.20
Baseline HbA_1c_, mean (SD)		11.43 (1.75)	12.05 (1.91)	11.72 (1.83)	.14

^a^ For MITI arm, n=32.

### Clinical Outcomes

#### Primary Outcome: Reaching Optimal Insulin Dose

**Figure 2:**
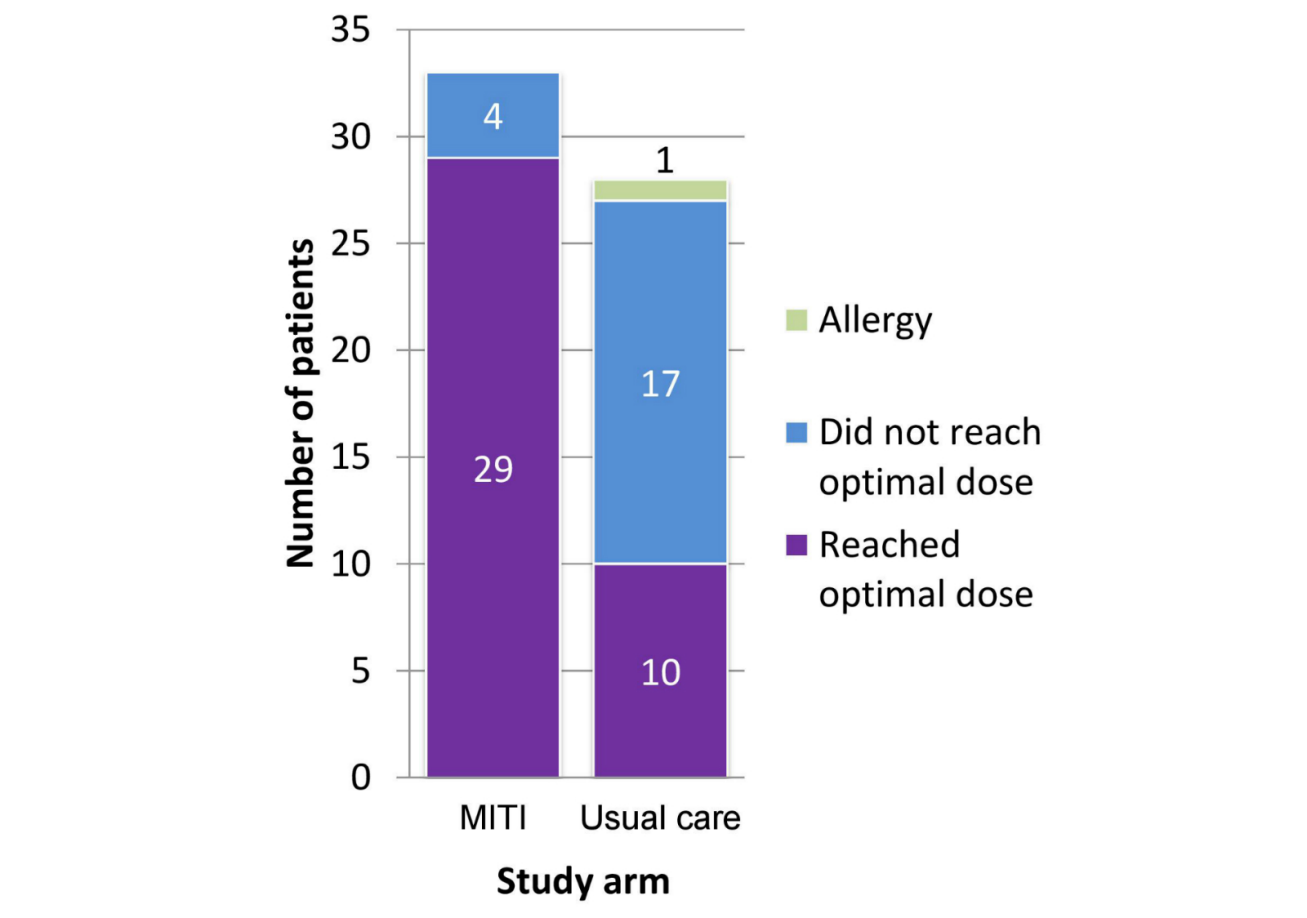
Number of patients who reached optimal insulin dose within 12 weeks. Note: for patients that did not reach optimal dose, 3 of 4 patients in the MITI arm and 1 of 17 in the usual care arm did not receive the allocated intervention.

The primary outcome was the number of patients that reached their optimal insulin glargine dose within 12 weeks. In the MITI arm, 29 of 33 patients (88%, 95% CI 72%-97%) reached their optimal dose. Of the 29 patients who met optimal dose, 27 did so by achieving a fasting blood glucose value between 80 and 130 mg/dL (inclusive). Two patients reached the maximum dose that could be safely administered. In the usual care arm, 10 of 27 patients (37%, 95% CI 19%-58%) reached their optimal dose (Figure 2). Of the 10 patients in the usual care arm that reached their optimal dose, 9 did so by achieving a fasting blood glucose between 80 and 130 mg/dL. One patient met this goal by reaching the maximum dose that could safely be administered. The primary outcome could not be measured for one usual care patient who discontinued insulin glargine early due to a possible allergic reaction. The MITI arm had a significantly greater proportion of patients reach their optimal insulin glargine dose (OR 12.3, 95% CI 3.3-45.4, *P*<.001).

For the 29 patients in the MITI arm that reached their optimal insulin glargine dose, the median time to optimal dose was 3.00 weeks (IQR 1.29-4.86). For the 10 patients in the usual care arm that reached optimal dose, the median time was 7.07 weeks (IQR 2.96-9.61, *P*=.007).

#### Glycated Hemoglobin

We measured HbA_1c_ change from baseline to 12 weeks. We included HbA_1c_ values from routine blood tests drawn within 4 weeks of baseline (study enrollment date) and 12 weeks after the baseline HbA_1c_ test (± 4 weeks). Looking at the nonimputed dataset, the mean HbA_1c_ for the MITI arm was 11.30% (SD 1.79, n=30) at baseline and 9.34% (SD 1.45, n=28) at 12 weeks. For the usual care arm, the mean was 12.20% (SD 1.90, n=25) at baseline and 9.99% (SD 1.33, n=14) at 12 weeks. The mean change for patients with an HbA_1c_ value at both baseline and 12 weeks was calculated. There were 28 patients in the MITI arm and 14 in the usual care arm. The mean change in HbA_1c_ between baseline and 12 weeks for the MITI arm was –1.90 (SD 2.64, n=28) and –1.81 (SD 2.63, n=14) for the usual care arm (*P*=.99). Combining the results from 10 multiple imputations (monotone method used), HbA_1c_ values in the MITI arm were 0.85 points lower than the usual care arm at 12 weeks (95% CI –1.83 to 0.13, *P*=.09). The large difference between the raw result and the multiple imputation result indicates that the missing mechanism was missing-not-at-random and the missing data problem was a limitation of this study for examining HbA_1c_ change.

#### Adverse Outcomes

There were 5 cases of hypoglycemia; 3 patients in the MITI arm and 2 in the usual care arm. All cases were mild with blood glucose values ranging between 69 and 79 mg/dL and none of the patients required assistance. One patient had a potential mild allergic reaction to insulin glargine.

#### Feasibility Outcomes

##### Text Message Responses

Of the 498 SMS text messages that were successfully sent to patients asking for their fasting blood glucose level, 420 (84.3%) received a reply. The GEE-adjusted response rate was 91.6% after adjusting for the difference in the number of text messages sent to/from patients and the correlation between responses. The mean number of SMS text messages successfully sent per patient was 18 texts (range 2-60). One patient did not receive the text prompts but was able to send back glucose values via text. The mean number of patient replies received was 16 texts (range 0-57). One patient who did receive the texts attempted to reply, but was not able to send the texts.

##### Titration Phone Calls

We reviewed each week that the nurse was assigned to call patients for their titration phone call (99 assigned calls total). The nurse was able to reach patients on the first or second attempt or by voicemail 91% of the time (90/99 assigned calls).

##### Time Spent by the Nurse on the Intervention

We reviewed the amount of time the diabetes nurse educator spent on the intervention. The vast majority of days (unless there was a technical issue that delayed Internet access), it took the nurse 1 minute or less to review texted blood glucose values on the Web portal. On titration Thursdays, the nurse spent approximately 7-11 minutes per patient. When assigned to call one patient, she spent a mean total of 11.2 minutes (SD 7.4) making phone calls. She spent a mean total of 17.6 (SD 10.9) minutes with 2 patients, 26.5 minutes (SD 14.5) with 3 patients, 36.2 minutes (SD 7.5) with 4 patients, and 36.0 minutes (SD 9.0) with 5 patients.

#### Cost Savings Outcomes

We recorded the number of titration interactions (any interaction with a clinician to address insulin dosage either by phone/voicemail or in-person). The MITI arm had 131 interactions: 75.6% (99/131) by phone/voicemail and 24.4% (32/131) in-person. The usual care arm had 49 interactions: 98.0% (48/49) were in-person and 2.0% (1/49) by phone. Looking only at those patients who received the allocated intervention, the MITI arm had a median of 3.5 (IQR 2.0-5.0) titration interactions and usual care had 2.0 (IQR 1.0-3.0, *P*=.003). As expected, MITI patients had more titration interactions by phone than in-person. The median number of phone interactions was 3.0 (IQR 1.0-5.0) for MITI. Only one patient in the usual care arm had a phone interaction occur during the study. The median number of in-person titration interactions for MITI was 1.0 (IQR 1.0-1.0) and 2.0 (IQR 1.0-3.0) for usual care (*P*=.009).

We also recorded the duration of titration interactions and compared the duration by type of interaction (not by study arm). The median duration of titration interactions in the clinic was 30.0 minutes (IQR 20.0-45.0). The median for phone/voicemail interactions was 6.0 minutes (IQR 3.0-10.0). The difference in duration between clinic and phone/voicemail interactions was statistically significant (*P*=.008).

We looked at patient utilization of the Bellevue health care system for appointments other than insulin titration (walk-in, emergency department, and medication refill visits). The MITI arm had no increased utilization of the health care system over the 12 weeks.

We asked our study participants in both arms if they had copays for appointments at our clinic. Of 61 participants, 37 (61%) reported that they had copays. Of those participants, 32 (86%) reported a copay of US $15.

We asked study participants in both arms about their travel time and wait time when they visited the clinic. Participants reported a median travel time to the clinic (1-way) of 45 minutes (IQR 30-60). The median wait time prior to appointments was 39 minutes (IQR 30-64).

#### Satisfaction Outcomes

The Diabetes Treatment Satisfaction Questionnaire status version has 6 questions that assess patient satisfaction with diabetes treatment using a 0-6 scale (0 being very dissatisfied and 6 being very satisfied). At the time of enrollment, the mean score for the MITI arm was 4.99 (SD 1.14, n=32) and 5.20 (SD 0.61, n=28) for the usual care arm (*P*=.78). At approximately 12 weeks after enrollment, the mean for the MITI arm was 5.74 (SD 0.54, n=27) and 5.53 (SD 0.52, n=22) for the usual care arm (*P*=.04). The mean difference between the baseline and 12-week scores for the MITI arm (n=27) was 0.80 and 0.34 for the usual care arm (n=22, *P*=.16).

The Diabetes Treatment Satisfaction Questionnaire Change version has 6 questions that assess the change in patient satisfaction over the course of the study using a –3 to +3 scale (–3 being much less satisfied now and +3 being much more satisfied now). The mean change score for MITI was 2.71 (SD 0.71) and 2.42 (SD 0.95) for the usual care arm (*P*=.13).

Patients responded to the 12-week follow-up questionnaires within a mean of 1.4 weeks (SD 1.5) and median of 0.8 (IQR 0.0-2.0) weeks before or after the 12-week date. The follow-up time for the satisfaction questionnaires ranged from 3.1 weeks before to 6.1 weeks after the 12-week date.

**Figure 3:**
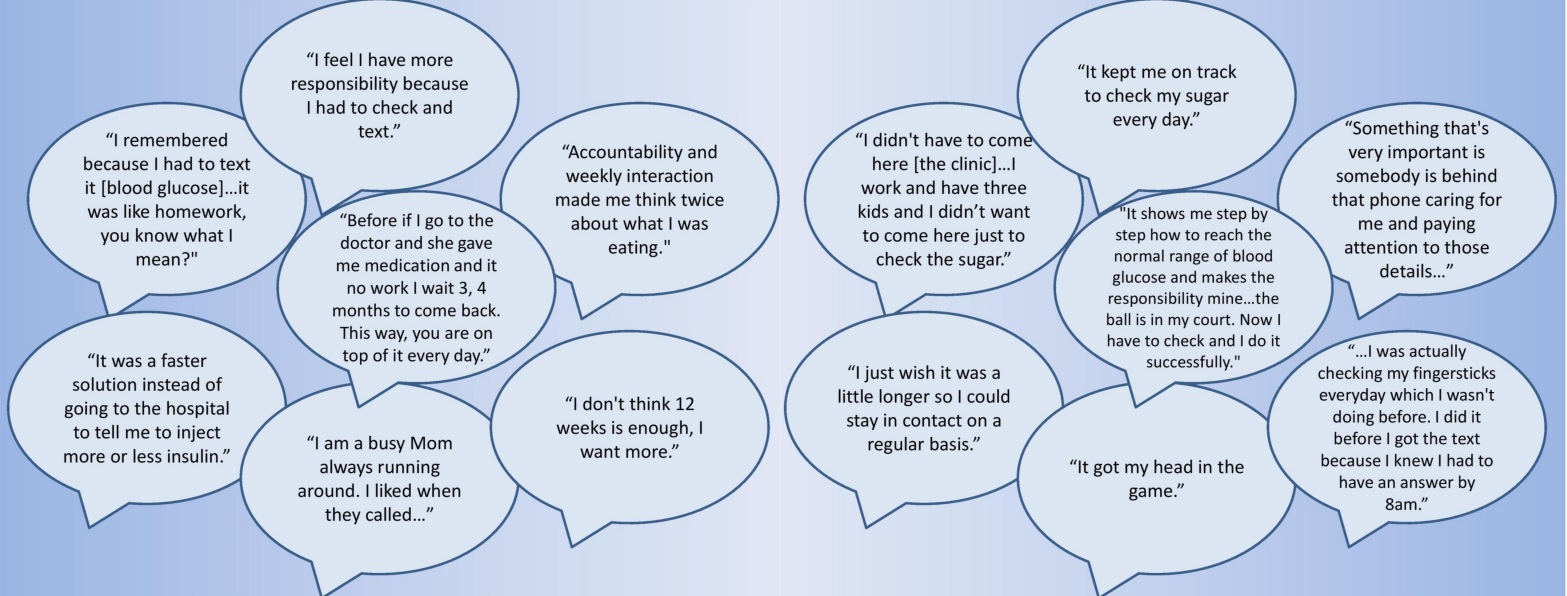
Patient feedback on the intervention.

A total of 27 patients in the MITI arm offered qualitative feedback on the intervention. Figure 3 shows selected quotes (from 11 patients) transcribed from the interview. The 14 quotes included in the figure are among the most specific comments made by patients about their experiences in the intervention.

## Discussion

### Principal Findings

#### Clinical Outcomes

Our study showed that with simple SMS text messaging (requiring only basic mobile phone technology) and weekly titration phone calls, 88% of diabetic patients reach their optimal insulin glargine dose within 12 weeks (vs 37% in usual care, *P*<.001). This outcome was achieved without an increase in hypoglycemia.

#### Feasibility Outcomes

Our study sent a daily SMS text message asking for a reply text with the morning’s fasting blood glucose and 84.3% of our texts received a reply. Our diabetes study nurse was able to relay titration instructions via phone calls for 91% of the assigned titration calls (within 2 tries or with a voicemail). Taken together, the response rates to our SMS text messages and the ease of reaching patients by phone support the feasibility of this intervention.

#### Cost Savings Outcomes

Patients enrolled in the MITI (texting) arm of our study saved travel time to and from the clinic, time spent in the waiting room, and the cost of copays, which 61% are required to pay at clinic appointments.

#### Satisfaction Outcomes

Through the Diabetes Treatment Satisfaction Questionnaire, we learned that MITI patients at 12 weeks were more satisfied with their treatment than usual care patients. The 14 quotes included in Figure 3 illustrate what some of our patients liked about their treatment during the intervention. These common themes include:

1. Text messages served as helpful reminders to check their glucose each morning.
2. Patients found the MITI intervention convenient.
3. Text messages made patients feel supported and cared for.
4. Patients liked knowing in a timely manner whether their insulin dose was adequate.
5. The need to check their blood glucose and report the values made patients feel more accountable for what they were eating and for taking their medications.

### Limitations

The generalizability of this study is limited for several reasons. With limited manpower for patient recruitment and enrollment, we were not able to meet our target sample size of 49 patients per arm. Voluntary participants may not be representative of the clinic population as a whole. We do not know if the gains of the intervention (the motivation to be more compliant with diet, exercise, daily insulin use, home glucose monitoring, etc) lasted beyond the 12 weeks of the study. Missing data (50% of usual care patients did not have a 12-week HbA_1c_ test) was a limitation in examining change in HbA_1c_. Patients must travel to the clinic to receive an HbA_1c_ blood test, which is a potential reason for the lack of HbA_1c_ data.

### Conclusions

MITI was shown to be a highly effective way to titrate insulin glargine. We used daily automated SMS text messages (simple mobile phone technology, no app required) to gather fasting blood glucose values. We used weekly nurse phone calls (based on a physician-approved algorithm) to deliver titration advice. This intervention was well-suited to our urban clinic population. MITI was feasible, time saving, and satisfactory. MITI should be implemented with a larger sample of patients to further test its effectiveness. Similar intervention models should be explored for other diabetic medication titrations as well as other challenging aspects of chronic disease care.

## Appendix

Multimedia Appendix 1 CONSORT-EHEALTH checklist V1.6.2 [30].

## Abbreviations

APCC: Adult Primary Care Center
HHC: Health and Hospitals Corporation
HbA1c: glycated hemoglobin
MITI: Mobile Insulin Titration Intervention
RA: research assistant
SES: socioeconomic status
SMS: short message service
