# Supplementary material for: The Mobile Insulin Titration Intervention (MITI) for Insulin Adjustment in an Urban, Low-Income Population: Randomized Controlled Trial
Source: J Med Internet Res. 2015 Jul 17;17(7):e180. doi: 10.2196/jmir.4716 (PMC4527003; doi:10.2196/jmir.4716)
Supplement: Multimedia Appendix 1 [file jmir_v17i7e180_app1.pdf]

CONSORT-EHEALTH Checklist V1.6.2 Report

(based on CONSORT-EHEALTH V1.6), available at [http://tinyurl.com/consort-ehealth-v1-6].

4716

Date completed

7/17/2015 10:28:14

by

Victoria Moynihan

The Mobile Insulin Titration Intervention (MITI) for Insulin Glargine Adjustment in an Urban, Low-Income Population: Randomized Controlled Trial

TITLE

1a-i) Identify the mode of delivery in the title

“The Mobile Insulin Titration Intervention (MITI) for Insulin Glargine Adjustment in an Urban, Low-Income Population: Randomized Controlled Trial”

1a-ii) Non-web-based components or important co-interventions in title

1a-iii) Primary condition or target group in the title

“The Mobile Insulin Titration Intervention (MITI) for Insulin Glargine Adjustment in an Urban, Low-Income Population: Randomized Controlled Trial”

ABSTRACT

1b-i) Key features/functionalities/components of the intervention and comparator in the METHODS section of the ABSTRACT

“Methods: This was a pilot study evaluating an intervention for patients requiring insulin glargine titration in the outpatient medical clinic of Bellevue Hospital Center in New York City. Patients in the intervention arm received weekday text messages from a health management platform requesting their fasting blood glucose values. The clinic’s diabetes nurse educator monitored the texted responses on the platform website each weekday for alarm values. Once a week, the nurse reviewed the glucose values, consulted the MITI titration algorithm, and called patients to adjust their insulin dose. Patients in the usual care arm continued to receive their standard clinic care for insulin titration. The primary outcome was whether a patient reached his/her optimal insulin glargine dose within 12 weeks.”

1b-ii) Level of human involvement in the METHODS section of the ABSTRACT

1b-iii) Open vs. closed, web-based (self-assessment) vs. face-to-face assessments in the METHODS section of the ABSTRACT

1b-iv) RESULTS section in abstract must contain use data

1b-v) CONCLUSIONS/DISCUSSION in abstract for negative trials

INTRODUCTION

2a-i) Problem and the type of system/solution

“Background
Many patients with diabetes mellitus in the United States are poorly controlled (glycated hemoglobin A1c [HbA1c] ≥9%). This includes 48.7% of the diabetics insured by Medicaid and 27.3% of diabetics insured by Medicare [1]. The consequences of uncontrolled diabetes are severe (eg, stroke, blindness, kidney disease, and amputation) and disproportionately affect patients of low socioeconomic status (SES) [2,3]. Insulin is commonly prescribed to treat uncontrolled diabetes [4]. Patients are started on a low dose of insulin and their dose is adjusted or “titrated” according to their blood glucose levels. Adjustments are made until the patient reaches a dose that best controls their glucose levels. Insulin titration traditionally occurs during a face-to-face encounter with a clinician [5-9]. Patients show the clinician their blood glucose levels from at-home testing and then the clinician recommends an appropriate insulin titration. One titration is often not enough to achieve glycemic control, so patients need to return to the clinic for multiple appointments.
Attending multiple appointments can be challenging for low-SES patients. They are faced with competing priorities that can make the many self-care tasks of diabetes management overwhelming [10-14]. Attending a clinic appointment can mean missing work hours with an inflexible job, lost wages, copays, and arranging for childcare and transportation to the clinic. Given these challenges, the process of insulin titration and achieving glycemic control may be prolonged.
Mobile phones are increasingly used to deliver health services [15]. Research shows 84% of the low-income US population owns a mobile phone, but only 47% of this population owns a phone with advanced features (ie, smartphone) [16]. Potential health interventions designed around basic features (eg, texting and voice) would not require a smartphone and, therefore, be the most accessible for low-income populations. In addition, these technologies still allow patients and clinicians to consult one another directly, allowing for personalized, nuanced care. A recent study of smartphone apps with insulin dose calculators showed that most have significant shortcomings. These apps may not take into account the patient’s level of clinical knowledge, missing glucose readings, or concurrent oral antihyperglycemic medications, potentially introducing a safety risk [17].”
“Current Intervention
The Mobile Insulin Titration Intervention (MITI) is a randomized controlled trial for patients who require insulin glargine titration. We chose to focus on glargine because it is the type of insulin used in our hospital formulary. Our intervention uses features available on basic mobile phones: text messaging and voice calls. This technology is easy to use, low-cost, and widely available to our patient population. Through text message, we can remind patients to check their glucose at any time and place that they have phone service. Patients can respond via text-quick and simple. Using weekly phone calls, patients and clinicians can still discuss their insulin treatment in a personal manner without the burden of an in-person appointment.
Through the MITI study, we aimed to (1) determine if MITI is effective in helping patients reach their optimal dose of insulin glargine (“optimal dose” is defined in the Intervention section), (2) evaluate the feasibility of the intervention, (3) measure the cost savings associated with the intervention, and (4) measure patient satisfaction.”

2a-ii) Scientific background, rationale: What is known about the (type of) system

“Mobile phones are increasingly used to deliver health services [15]. Research shows 84% of the low-income US population owns a mobile phone, but only 47% of this population owns a phone with advanced features (ie, smartphone) [16]. Potential health interventions designed around basic features (eg, texting and voice) would not require a smartphone and, therefore, be the most accessible for low-income populations. In addition, these technologies still allow patients and clinicians to consult one another directly, allowing for personalized, nuanced care. A recent study of smartphone apps with insulin dose calculators showed that most have significant shortcomings. These apps may not take into account the patient’s level of clinical knowledge, missing glucose readings, or concurrent oral antihyperglycemic medications, potentially introducing a safety risk [17].”
“Prior Research
Studies show that text messaging is an effective medium to assist with diabetes management in general and low-SES populations [6,18-25]. It can be used successfully to remind patients to check their blood glucose levels and to gather that data so that a clinician can review it for the next in-person clinic appointment [19].
Of the few studies in which clinicians titrated insulin remotely, the interventions typically required Internet access or website navigation. Patients sent their blood glucose values to their clinicians via the Internet. Clinicians responded to these data by sending their recommendations over the Internet or by short message service (SMS) text message. These studies show that it is feasible to have patients send their blood glucose data and have clinicians relay insulin dose titration advice remotely [6,22,26]. However, with our intervention we aim to show that this exchange of data can be achieved using only basic text message and voice technology.”

METHODS

3a) CONSORT: Description of trial design (such as parallel, factorial) including allocation ratio
“Through the MITI study, we aimed to (1) determine if MITI is effective in helping patients reach their optimal dose of insulin glargine (“optimal dose” is defined in the Intervention section), (2) evaluate the feasibility of the intervention, (3) measure the cost savings associated with the intervention, and (4) measure patient satisfaction.”

3b) CONSORT: Important changes to methods after trial commencement (such as eligibility criteria), with reasons
“Implementation Challenges
Our initial health management platform was not able to send text messages to patients with prepaid phones; thus, these patients were not able to sign up to participate in the intervention (see Participant Characteristics). These patients continued to attend in-person appointments for insulin titration and were included in the intention-to-treat analysis. Subsequent patients with incompatible phones were provided a mobile phone to use during the study. Beginning in May 2014, patients were enrolled using a different health management platform that accommodated all types of mobile phones.
We initially stratified participants by insulin treatment status (initiating insulin or needing their existing dose titrated) and by HbA1c level (8%-11% or >11%). In May 2014, we decided to stratify only by insulin treatment status after finding that not all participants had an HbA1c value in their medical record on the day of study enrollment.”

3b-i) Bug fixes, Downtimes, Content Changes

4a) CONSORT: Eligibility criteria for participants
“Inclusion and Exclusion Criteria
The inclusion criteria for patients were initiating insulin glargine or requiring the titration of an existing insulin glargine dose, English or Spanish speaking, the most recent HbA1c value at or above 8%, able and willing to inject insulin, and able and willing to provide informed consent. The exclusion criteria were patients on short-acting insulin, on systemic glucocorticoids, with sustained serum creatinine at or above 1.5 mg/dL for men and 1.4 mg/dL for women, with documented hypoglycemia unawareness, and with type 1 diabetes.”

4a-i) Computer / Internet literacy

4a-ii) Open vs. closed, web-based vs. face-to-face assessments:
“The RA screened patients for eligibility and enrolled them in the study. The enrollment process occurred in-person in the clinic and all patients provided informed consent before participating in the study.”
“Intervention
After consent and randomization, patients in the MITI arm enrolled in a Web-based health management platform during the enrollment process at the clinic. The platform automatically sent patients a text message each weekday morning asking them for their fasting blood glucose value. During enrollment, the patient was able to choose English or Spanish messages and the specific time of day when the messages would be sent. When patients received the text message on their phone, they responded with their blood glucose value. The diabetes nurse educator logged onto her secure account on the platform each weekday afternoon to view the patients’ text message responses. She would call any patient that had texted an alarm value (blood glucose <80 or >400 mg/dL). Patients were instructed to call the diabetes nurse educator (which is the standard practice with patients in the clinic) in addition to sending the text if they had an alarm value.
Each Thursday afternoon, the nurse reviewed the texted values, consulted the titration algorithm (which was developed by physicians and nurses on the study team), and called the patient to adjust his/her insulin dose. The nurse could call the patient’s emergency contact on her discretion. Beginning in May 2014, we revised our study protocol and outlined voicemail as an option for the nurse to give titration instructions to patients. When the nurse was not available to check the text responses for alarm values or to make titration calls, a physician on the study team performed this task.
Patients continued with the weekday text messages and weekly phone calls until they reached their optimal insulin glargine dose or for a maximum of 12 weeks. We defined optimal insulin dose as the dose at which a patient achieved at least 1 fasting blood glucose value between 80 and 130 mg/dL (inclusive) or the maximum dose that could be safely administered to the patient. During the intervention, patients continued to attend appointments with their primary care provider, but did not need to attend appointments specifically for diabetes management (eg, high HbA1c clinic or diabetes nurse educator appointments). After completing the intervention, patients resumed usual clinic care. The study team arranged any follow-up appointments needed to allow the patient to resume their standard diabetes care (eg, primary care provider, diabetes nurse educator, and high HbA1c clinic appointments).”
“Usual Care
After consent and randomization, patients were instructed to continue with their existing treatment plan and appointments for diabetes care. After the patient had a clinic appointment for insulin titration, the RA collected data (in-person or by phone) about the appointment. These data included the patient’s insulin dose, blood glucose values, and data for cost savings outcomes.”
“Follow-Up
At approximately 12 weeks after study enrollment, patients in both arms were contacted by the RA to remind them of their routine HbA1c test and to ask them to fill out the Diabetes Treatment Satisfaction Questionnaire (either over the phone or when the patient was in the clinic).”

4a-iii) Information giving during recruitment

4b) CONSORT: Settings and locations where the data were collected
“The enrollment process occurred in-person in the clinic and all patients provided informed consent before participating in the study.”
“The diabetes nurse educator logged onto her secure account on the platform each weekday afternoon to view the patients’ text message responses.”
“After the patient had a clinic appointment for insulin titration, the RA collected data (in-person or by phone) about the appointment.”
“At approximately 12 weeks after study enrollment, patients in both arms were contacted by the RA to remind them of their routine HbA1c test and to ask them to fill out the Diabetes Treatment Satisfaction Questionnaire (either over the phone or when the patient was in the clinic).”

4b-i) Report if outcomes were (self-)assessed through online questionnaires

Outcomes were not assessed through online questionnaires in this study.

4b-ii) Report how institutional affiliations are displayed

5) CONSORT: Describe the interventions for each group with sufficient details to allow replication, including how and when they were actually administered

5-i) Mention names, credential, affiliations of the developers, sponsors, and owners

5-ii) Describe the history/development process

5-iii) Revisions and updating

5-iv) Quality assurance methods

5-v) Ensure replicability by publishing the source code, and/or providing screenshots/screen-capture video, and/or providing flowcharts of the algorithms used

5-vi) Digital preservation

5-vii) Access
"The enrollment process occurred in-person in the clinic and all patients provided informed consent before participating in the study."
"After consent and randomization, patients in the MITI arm enrolled in a Web-based health management platform during the enrollment process at the clinic."

5-viii) Mode of delivery, features/functionalities/components of the intervention and comparator, and the theoretical framework
"Intervention
After consent and randomization, patients in the MITI arm enrolled in a Web-based health management platform during the enrollment process at the clinic. The platform automatically sent patients a text message each weekday morning asking them for their fasting blood glucose value. During enrollment, the patient was able to choose English or Spanish messages and the specific time of day when the messages would be sent. When patients received the text message on their phone, they responded with their blood glucose value. The diabetes nurse educator logged onto her secure account on the platform each weekday afternoon to view the patients' text message responses."

5-ix) Describe use parameters

5-x) Clarify the level of human involvement

5-xi) Report any prompts/reminders used
"The platform automatically sent patients a text message each weekday morning asking them for their fasting blood glucose value. During enrollment, the patient was able to choose English or Spanish messages and the specific time of day when the messages would be sent. When patients received the text message on their phone, they responded with their blood glucose value."

5-xii) Describe any co-interventions (incl. training/support)

This study does not include any co-interventions.

6a) CONSORT: Completely defined pre-specified primary and secondary outcome measures, including how and when they were assessed
"After the patient had a clinic appointment for insulin titration, the RA collected data (in-person or by phone) about the appointment. These data included the patient's insulin dose, blood glucose values, and data for cost savings outcomes."
"Outcome Measures
Our primary outcome was whether a patient reached his/her optimal insulin glargine dose within 12 weeks of enrolling in the study. We hypothesized that a greater proportion of patients in the MITI arm would reach their optimal insulin dose as compared to the usual care arm. The research staff recorded whether a patient reached his/her optimal insulin dose after each titration phone call (for the MITI arm) or after each clinic appointment (for the usual care arm). We also examined the time it took to reach optimal dose, patient self-reported hypoglycemia, and change in HbA1c levels between baseline and 12 weeks.
We measured the feasibility of the intervention, including patient text response rate, the ability of the nurse to reach patients for titration phone calls, and the time the nurse spent on the intervention.
We collected data on the cost savings associated with the intervention. These data included the number and duration of insulin titration interactions (appointments during which insulin was titrated), the time patients spent traveling to the clinic and waiting prior to appointments, copays for clinic appointments, and patient health care utilization (the number of walk-in clinic, medication refill, and emergency room visits at Bellevue Hospital Center). Copays refer to the amount that the patient pays the clinic on attending an appointment with a health care provider. For patients with insurance plans, the amount is typically set by the insurance company. For uninsured patients at our hospital, the amount is based on income. For patients in our study, the most common copay was US $15.
To assess patient satisfaction with the intervention, we used the Diabetes Treatment Satisfaction Questionnaire (status version) [29]. This was administered at study enrollment and approximately 12 weeks later. We also administered the Diabetes Treatment Satisfaction Questionnaire (change version) [29] to measure the change in satisfaction after study participation. Patients in the MITI arm participated in a semistructured interview to give qualitative feedback on the intervention. This occurred when the patient reached his/her optimal dose or when the 12 weeks had elapsed."

6a-i) Online questionnaires: describe if they were validated for online use and apply CHERRIES items to describe how the questionnaires were designed/deployed

6a-ii) Describe whether and how "use" (including intensity of use/dosage) was defined/measured/monitored

6a-iii) Describe whether, how, and when qualitative feedback from participants was obtained

6b) CONSORT: Any changes to trial outcomes after the trial commenced, with reasons

Qualitative feedback interview was added as an outcome.

7a) CONSORT: How sample size was determined

7a-i) Describe whether and how expected attrition was taken into account when calculating the sample size

7b) CONSORT: When applicable, explanation of any interim analyses and stopping guidelines
"A data and safety monitoring board reviewed any potential safety concerns for the duration of the study. This manuscript summarizes the methods for this trial."

8a) CONSORT: Method used to generate the random allocation sequence
"The random allocation sequence was computer-generated by a coinvestigator and concealed in sequentially numbered envelopes. Patients were stratified by whether they were initiating insulin treatment or having their existing insulin dose adjusted. Within each stratification, the allocation sequence used blocks of 4 to help keep the number of patients balanced in each arm."

8b) CONSORT: Type of randomisation; details of any restriction (such as blocking and block size)
"The random allocation sequence was computer-generated by a coinvestigator and concealed in sequentially numbered envelopes. Patients were stratified by whether they were initiating insulin treatment or having their existing insulin dose adjusted. Within each stratification, the allocation sequence used blocks of 4 to help keep the number of patients balanced in each arm."

9) CONSORT: Mechanism used to implement the random allocation sequence (such as sequentially numbered containers), describing any steps taken to conceal the sequence until interventions were assigned
"The random allocation sequence was computer-generated by a coinvestigator and concealed in sequentially numbered envelopes. Patients were stratified by whether they were initiating insulin treatment or having their existing insulin dose adjusted. Within each stratification, the allocation sequence used blocks of 4 to help keep the number of patients balanced in each arm."

10) CONSORT: Who generated the random allocation sequence, who enrolled participants, and who assigned participants to interventions
"The random allocation sequence was computer-generated by a coinvestigator and concealed in sequentially numbered envelopes. Patients were stratified by whether they were initiating insulin treatment or having their existing insulin dose adjusted. Within each stratification, the allocation sequence used blocks of 4 to help keep the number of patients balanced in each arm."
"The RA screened patients for eligibility and enrolled them in the study. The enrollment process occurred in person in the clinic and all patients provided informed consent before to participating in the study."
The RA assigned participants to the interventions during the enrollment process.

11a) CONSORT: Blinding - If done, who was blinded after assignment to interventions (for example, participants, care providers, those assessing outcomes) and how

11a-i) Specify who was blinded, and who wasn't

"Patients, clinicians, and researchers in this trial were not blinded to arm assignments."

11a-ii) Discuss e.g., whether participants knew which intervention was the "intervention of interest" and which one was the "comparator"

11b) CONSORT: If relevant, description of the similarity of interventions

This item was not relevant to this trial.

12a) CONSORT: Statistical methods used to compare groups for primary and secondary outcomes
"Statistical Analysis
Baseline characteristics were summarized using descriptive statistics and compared to determine if the arms were balanced. Chi-square tests were used for categorical outcomes and Wilcoxon rank sum tests for continuous outcomes. Interval-censoring survival analysis was used to analyze the time to reach optimal insulin dose. The generalized estimating equation (GEE) modeling was used for repeatedly measured text message responses and the duration of titration interactions. Multiple imputation was used to deal with missing data in HbA1c measures. Intention-to-treat analysis was used."

12a-i) Imputation techniques to deal with attrition / missing values

"Multiple-imputation was used to deal with missing data in A1c measures."

12b) CONSORT: Methods for additional analyses, such as subgroup analyses and adjusted analyses
"Interval-censoring survival analysis was used to analyze the time to reach optimal insulin dose. The generalized-estimating-equation (GEE) modeling was used for repeatedly measured text message responses and the duration of titration interactions. Multiple-imputation was used to deal with missing data in A1c measures."

RESULTS

13a) CONSORT: For each group, the numbers of participants who were randomly assigned, received intended treatment, and were analysed for the primary outcome
"Primary Outcome: Reaching Optimal Insulin Dose
The primary outcome was the number of patients that reached their optimal insulin glargine dose within 12 weeks. In the MITI arm, 29 of 33 patients (88%, 95% CI 72%-97%) reached their optimal dose. Of the 29 patients who met optimal dose, 27 did so by achieving a fasting blood glucose value between 80 and 130 mg/dL (inclusive). Two patients reached the maximum dose that could be safely administered. In the usual care arm, 10 of 27 patients (37%, 95% CI 19%-58%) reached their optimal dose (Figure 2). Of the 10 patients in the usual care arm that reached their optimal dose, 9 did so by achieving a fasting blood glucose between 80 and 130 mg/dL. One patient met this goal by reaching the maximum dose that could safely be administered. The primary outcome could not be measured for 1 usual care patient who discontinued insulin glargine early due to a possible allergic reaction. The MITI arm had a significantly greater proportion of patients reach their optimal insulin glargine dose (OR 12.3, 95% CI 3.3-45.4, P<.001)."

13b) CONSORT: For each group, losses and exclusions after randomisation, together with reasons

Please see CONSORT diagram

13b-i) Attrition diagram

14a) CONSORT: Dates defining the periods of recruitment and follow-up

"Participant characteristics
Patients were recruited from June 2013 to December 2013 and May 2014 to December 2014. Follow-up data was collected until March 2015."

14a-i) Indicate if critical "secular events" fell into the study period

14b) CONSORT: Why the trial ended or was stopped (early)

This trial did not end early.

15) CONSORT: A table showing baseline demographic and clinical characteristics for each group
Please see table 1.

15-i) Report demographics associated with digital divide issues

Please see table 1.

16a) CONSORT: For each group, number of participants (denominator) included in each analysis and whether the analysis was by original assigned groups

16-i) Report multiple "denominators" and provide definitions
"Primary Outcome: Reaching Optimal Insulin Dose
The primary outcome was the number of patients that reached their optimal insulin glargine dose within 12 weeks. In the MITI arm, 29 of 33 patients (88%, 95% CI 72%-97%) reached their optimal dose. Of the 29 patients who met optimal dose, 27 did so by achieving a fasting blood glucose value between 80 and 130 mg/dL (inclusive). Two patients reached the maximum dose that could be safely administered. In the usual care arm, 10 of 27 patients (37%, 95% CI 19%-58%) reached their optimal dose (Figure 2). Of the 10 patients in the usual care arm that reached their optimal dose, 9 did so by achieving a fasting blood glucose between 80 and 130 mg/dL. One patient met this goal by reaching the maximum dose that could safely be administered. The primary outcome could not be measured for 1 usual care patient who discontinued insulin glargine early due to a possible allergic reaction. The MITI arm had a significantly greater proportion of patients reach their optimal insulin glargine dose (OR 12.3, 95% CI 3.3-45.4, P<.001).
For the 29 patients in the MITI arm that reached their optimal insulin glargine dose, the median time to optimal dose was 3.00 weeks (IQR 1.29-4.86).
For the 10 patients in the usual care arm that reached optimal dose, the median time was 7.07 weeks (IQR 2.96-9.61, P=.007)."

16-ii) Primary analysis should be intent-to-treat

17a) CONSORT: For each primary and secondary outcome, results for each group, and the estimated effect size and its precision (such as 95% confidence interval)

"Primary Outcome: Reaching Optimal Insulin Dose
The primary outcome was the number of patients that reached their optimal insulin glargine dose within 12 weeks. In the MITI arm, 29 of 33 patients (88%, 95% CI 72%-97%) reached their optimal dose. Of the 29 patients who met optimal dose, 27 did so by achieving a fasting blood glucose value between 80 and 130 mg/dL (inclusive). Two patients reached the maximum dose that could be safely administered. In the usual care arm, 10 of 27 patients (37%, 95% CI 19%-58%) reached their optimal dose (Figure 2). Of the 10 patients in the usual care arm that reached their optimal dose, 9 did so by achieving a fasting blood glucose between 80 and 130 mg/dL. One patient met this goal by reaching the maximum dose that could safely be administered. The primary outcome could not be measured for 1 usual care patient who discontinued insulin glargine early due to a possible allergic reaction. The MITI arm had a significantly greater proportion of patients reach their optimal insulin glargine dose (OR 12.3, 95% CI 3.3-45.4, P<.001)."

"HbA1c
We measured HbA1c change from baseline to 12 weeks. We included HbA1c values from routine blood tests drawn within 4 weeks of baseline (study enrollment date) and 12 weeks after the baseline HbA1c test (+/- 4 weeks). Looking at the nonimputed dataset, the mean HbA1c for the MITI arm was 11.30% (SD 1.79, n=30) at baseline and 9.34% (SD 1.45, n=28) at 12 weeks. For the usual care arm, the mean was 12.20% (SD 1.90, n=25) at baseline and 9.99% (SD 1.33, n=14) at 12 weeks. The mean change for patients with an HbA1c value at both baseline and 12 weeks was calculated. There were 28 patients in the MITI group and 14 in the usual care group. The mean change in HbA1c between baseline and 12 weeks for the MITI arm was –1.90 (SD 2.64, n=28) and –1.81 (SD 2.63, n=14) for the usual care arm (P=.99). Combining the results from 10 multiple imputations (monotone method used), HbA1c values in the MITI arm were 0.85 points lower than the usual care arm at 12 weeks (95% CI –1.83 to 0.13, P=.09). The large difference between the raw result and the multiple imputation result indicates that the missing mechanism was missing-not-at-random and the missing data problem was a limitation of this study for examining HbA1c change."

17a-i) Presentation of process outcomes such as metrics of use and intensity of use

17b) CONSORT: For binary outcomes, presentation of both absolute and relative effect sizes is recommended

Not reported.

18) CONSORT: Results of any other analyses performed, including subgroup analyses and adjusted analyses, distinguishing pre-specified from exploratory

"The GEE-adjusted response rate was 91.6%, after adjusting for the difference in the number of text messages sent to/from patients and the correlation between responses."

"Looking only at those patients who received the allocated intervention, the MITI arm had a median of 3.5 (IQR 2.0-5.0) titration interactions and usual care had 2.0 (IQR 1.0-3.0, P=.003)."

18-i) Subgroup analysis of comparing only users

19) CONSORT: All important harms or unintended effects in each group

"Adverse Outcomes
There were five cases of hypoglycemia; three participants in the MITI arm and two in the usual-care arm. All cases were mild, with blood glucose values ranging between 69 and 79 mg/dL, and none of the participants required assistance. One patient had a potential mild allergic reaction to insulin glargine."

19-i) Include privacy breaches, technical problems

19-ii) Include qualitative feedback from participants or observations from staff/researchers

DISCUSSION

20) CONSORT: Trial limitations, addressing sources of potential bias, imprecision, multiplicity of analyses

20-i) Typical limitations in ehealth trials

"Limitations
The generalizability of this study is limited for several reasons. With limited manpower for patient recruitment and enrollment, we were not able to meet our target sample size of 49 patients per arm. Voluntary participants may not be representative of the clinic population as a whole. We do not know if the gains of the intervention (the motivation to be more compliant with diet, exercise, daily insulin use, home glucose monitoring, etc) lasted beyond the 12 weeks of the study. Missing data (50% of usual care patients did not have a 12-week HbA1c test) was a limitation in examining change in HbA1c. Patients must travel to the clinic to receive an HbA1c blood test, which is a potential reason for the lack of HbA1c data."

21) CONSORT: Generalisability (external validity, applicability) of the trial findings

21-i) Generalizability to other populations

21-ii) Discuss if there were elements in the RCT that would be different in a routine application setting

22) CONSORT: Interpretation consistent with results, balancing benefits and harms, and considering other relevant evidence

22-i) Restate study questions and summarize the answers suggested by the data, starting with primary outcomes and process outcomes (use)

"Clinical outcomes
Our study showed that with simple text messaging (requiring only basic cell phone technology)and weekly titration phone calls, 88% of diabetic patients reach their optimal insulin glargine dose within 12 weeks (vs. 37% in usual-care, P<.001). This outcome was achieved without an increase in hypoglycemia."

"Limitations
The generalizability of this study is limited for several reasons. With limited manpower for patient recruitment and enrollment, we were not able to meet our target sample size of 49 patients per arm. Voluntary participants may not be representative of the clinic population as a whole. We do not know if the gains of the intervention (the motivation to be more compliant with diet, exercise, daily insulin use, home glucose monitoring, etc) lasted beyond the 12 weeks of the study. Missing data (50% of usual care patients did not have a 12-week HbA1c test) was a limitation in examining change in HbA1c. Patients must travel to the clinic to receive an HbA1c blood test, which is a potential reason for the lack of HbA1c data."

22-ii) Highlight unanswered new questions, suggest future research

Other information

23) CONSORT: Registration number and name of trial registry

"Trial Registration: Clinicaltrials.gov NCT01879579; https://clinicaltrials.gov/ct2/show/NCT01879579"

24) CONSORT: Where the full trial protocol can be accessed, if available

Levy N, Moynihan V, Nilo A, Singer K, Bernik LS, Etiebet MA, Fang Y, Cho J, Natarajan S. The Mobile Insulin Titration Intervention (MITI) for Insulin Glargine Titration in an Urban, Low-Income Population: Randomized Controlled Trial Protocol. JMIR Res Protoc 2015;4(1):e31

URL: http://www.researchprotocols.org/2015/1/e31
DOI: 10.2196/resprot.4206
PMID: 25794243
PMCID: 4381814

25) CONSORT: Sources of funding and other support (such as supply of drugs), role of funders

"We would like to thank our funders, the New York University-Health and Hospitals Corporation Clinical and Translational Science Institute (NYU-HHC CTSI) for the 2013 NYU CTSI Pilot Grant and the 2014 HHC H-3 Research Grant award # UL1 TR000038 from the National Center for the Advancement of Translational Science (NCATS), National Institutes of Health."

X26-i) Comment on ethics committee approval

x26-ii) Outline informed consent procedures

X26-iii) Safety and security procedures

X27-i) State the relation of the study team towards the system being evaluated
